# Assessment of the capacity of vehicle cabin air inlet filters to reduce diesel exhaust-induced symptoms in human volunteers

**DOI:** 10.1186/1476-069X-13-16

**Published:** 2014-03-13

**Authors:** Ala Muala, Maria Sehlstedt, Anne Bion, Camilla Österlund, Jenny A Bosson, Annelie F Behndig, Jamshid Pourazar, Anders Bucht, Christoffer Boman, Ian S Mudway, Jeremy P Langrish, Stephane Couderc, Anders Blomberg, Thomas Sandström

**Affiliations:** 1 Department of Public Health and Clinical Medicine, Division of Medicine, Umeå University, Umeå, Sweden; 2 Renault Technocentre, Guyancourt, France; 3 Swedish Defence Research Agency, FOI, Umeå, Sweden; 4 Department of Applied Physics and Electronics, Thermochemical Energy Conversion Laboratory, Umeå University, Umeå, Sweden; 5 MRC-PHE Centre for Environment and Health, School of Biomedical Sciences, King’s College London, London, UK; 6 BHF/University Centre for Cardiovascular Science, University of Edinburgh, Edinburgh, UK

## Abstract

**Background:**

Exposure to particulate matter (PM) air pollution especially derived from traffic is associated with increases in cardiorespiratory morbidity and mortality. In this study, we evaluated the ability of novel vehicle cabin air inlet filters to reduce diesel exhaust (DE)-induced symptoms and markers of inflammation in human subjects.

**Methods:**

Thirty healthy subjects participated in a randomized double-blind controlled crossover study where they were exposed to filtered air, unfiltered DE and DE filtered through two selected particle filters, one with and one without active charcoal. Exposures lasted for one hour. Symptoms were assessed before and during exposures and lung function was measured before and after each exposure, with inflammation assessed in peripheral blood five hours after exposures. In parallel, PM were collected from unfiltered and filtered DE and assessed for their capacity to drive damaging oxidation reactions in a cell-free model, or promote inflammation in A549 cells.

**Results:**

The standard particle filter employed in this study reduced PM_10_ mass concentrations within the exposure chamber by 46%, further reduced to 74% by the inclusion of an active charcoal component. In addition use of the active charcoal filter was associated by a 75% and 50% reduction in NO_2_ and hydrocarbon concentrations, respectively. As expected, subjects reported more subjective symptoms after exposure to unfiltered DE compared to filtered air, which was significantly reduced by the filter with an active charcoal component. There were no significant changes in lung function after exposures. Similarly diesel exhaust did not elicit significant increases in any of the inflammatory markers examined in the peripheral blood samples 5 hour post-exposure. Whilst the filters reduced chamber particle concentrations, the oxidative activity of the particles themselves, did not change following filtration with either filter. In contrast, diesel exhaust PM passed through the active charcoal combination filter appeared less inflammatory to A549 cells.

**Conclusions:**

A cabin air inlet particle filter including an active charcoal component was highly effective in reducing both DE particulate and gaseous components, with reduced exhaust-induced symptoms in healthy volunteers. These data demonstrate the effectiveness of cabin filters to protect subjects travelling in vehicles from diesel exhaust emissions.

## Introduction

It is well documented that exposure to air pollution causes adverse cardiorespiratory health effects and a large number of epidemiological studies have documented the relationship between increased fine particulate air pollution and high mortality rates [1-3]. Exposure to traffic-derived particulate air pollution is associated with a deterioration of asthma in children and adults, chronic obstructive pulmonary disease (COPD) in the elderly [4-6], as well as increases in cardiovascular morbidity and mortality [7-10].

Diesel exhaust (DE) has been shown to be a predominant contributor to urban fine particulate matter contributing to adverse health effects [11]. Previous experimental human exposure studies have demonstrated that exposure to diesel engine exhaust induces a wide range of airway inflammatory responses including increased inflammatory cell infiltration along with an enhanced cytokines release through the activation of redox-sensitive transcription factors [12-14]. We have also previously demonstrated impaired vasomotor function and endogenous fibrinolysis, enhanced ex vivo thrombus formation and increase arterial stiffness in subjects following controlled diesel exhaust challenge [15-17]. Furthermore, we have demonstrated that exhaust particle traps not only reduce the emission particle mass and number but also prevent cardiovascular and prothrombotic effects [18].

Exposure to traffic-derived air pollution is therefore of major public health concern in urban areas, estimated by the WHO to result in 3.2 million deaths annually worldwide [19], and there is an urgent need to consider and evaluate strategies to reduce individual exposures [20]. Whilst the best strategy, with the widest possible benefit to the population, would be to reduce emissions though legislation, as was achieved for coal [21,22] and cigarette smoke [23,24], such interventions for traffic are challenged by economical and political difficulties. Other strategies are therefore required, either through improved tailpipe emissions abatement technologies, or traffic management schemes. In addition, the use of tail-pipe particle filters has been shown to reduce many of the adverse cardiovascular effects of diesel exhaust exposure, and the use of a highly efficient facemask in heavily polluted urban areas is associated with small improvements in blood pressure, heart rate variability, myocardial ischemia and respiratory symptoms [25,26]. However, much of an individual’s exposure to traffic-derived air pollution may actually be received whilst commuting in traffic within vehicles or walking or cycling in urban areas [6]. Recent studies have shown that exposure to PM within a car or bus is often 20-70% higher than for cyclists along a similar route [27,28]. Modern in-car filtration systems can actually reduce PM exposure within cars significantly [29], and similar filtration systems within people’s homes have been shown to both reduce exposure to PM and improve measures of microvascular function [30].

We therefore suggest that the approach to protect individuals from PM exposure is to target in-car exposures by means of highly efficient air inlet filters. This concept was previously investigated by employing cabin air filters in an experimental human diesel exhaust exposure study. It was demonstrated that particle filters with active charcoal significantly reduced symptoms [31]. The study led to the application of scientifically evaluated cabin air inlet filters by the vehicle industry.

The present investigation focuses on the efficacy of modern air inlet filters. We evaluated the efficacy and ability of a series of newly developed cabin air filters to decrease DE-induced respiratory symptoms in healthy volunteers. In addition, we also examined whether the filters altered the oxidative and pro-inflammatory potentials of the residual particles penetrating the filter to the breathing zone of the subjects. We hypothesized that reduction of PM mass, as well as other components of the diesel exhaust aerosol by cabin filters would reduce irritant symptoms and reduce adverse health effects.

The aim of this study was evaluate the efficacy of different vehicle cabin air inlet filters to reduce the diesel exhaust induced symptomatic responses in healthy subjects.

## Methods

### Subjects

Thirty healthy volunteers (mean age 25, range 18-29, 17 males, 13 females, all never smokers) were recruited. All subjects underwent a physical examination, baseline blood count and renal function assessment, spirometry (FEV_1_, VC, FEV_1_/VC) and 12-lead electrocardiogram prior to participation. All subjects completed a cardiopulmonary exercise test on an upright bicycle ergometer to determine the workload required to produce an average minute ventilation of 20 L/min/m^2^ body surface area. All subjects gave their written informed consent and the study was approved by the local ethical review board, and carried out in accordance with the Declaration of Helsinki.

### Exposures

Subjects were exposed separately to filtered air or diesel exhaust, filtered and unfiltered, on four separate occasions in a randomized double-blind controlled crossover manner (see Additional file 1: Figure S1 for graphical presentation). Filtration of the diesel exhaust was done with two separate filters; Filter A, which was a particle filter and Filter B, which was the same particle filter with an active charcoal filter medium added. The exposures were separated by at least one week. Each exposure lasted for one hour during which the subjects performed moderate physical exercise (minute ventilation 20 L/min/m^2^ body surface area) on a bicycle ergometer for 15 min followed by 15 min rest, repeated during the second half of the exposures [32]. During filtered air exposures, the engine was also running, but no exhaust was fed into the chamber.

### Diesel generation and particle characterization

**Figure 1:**
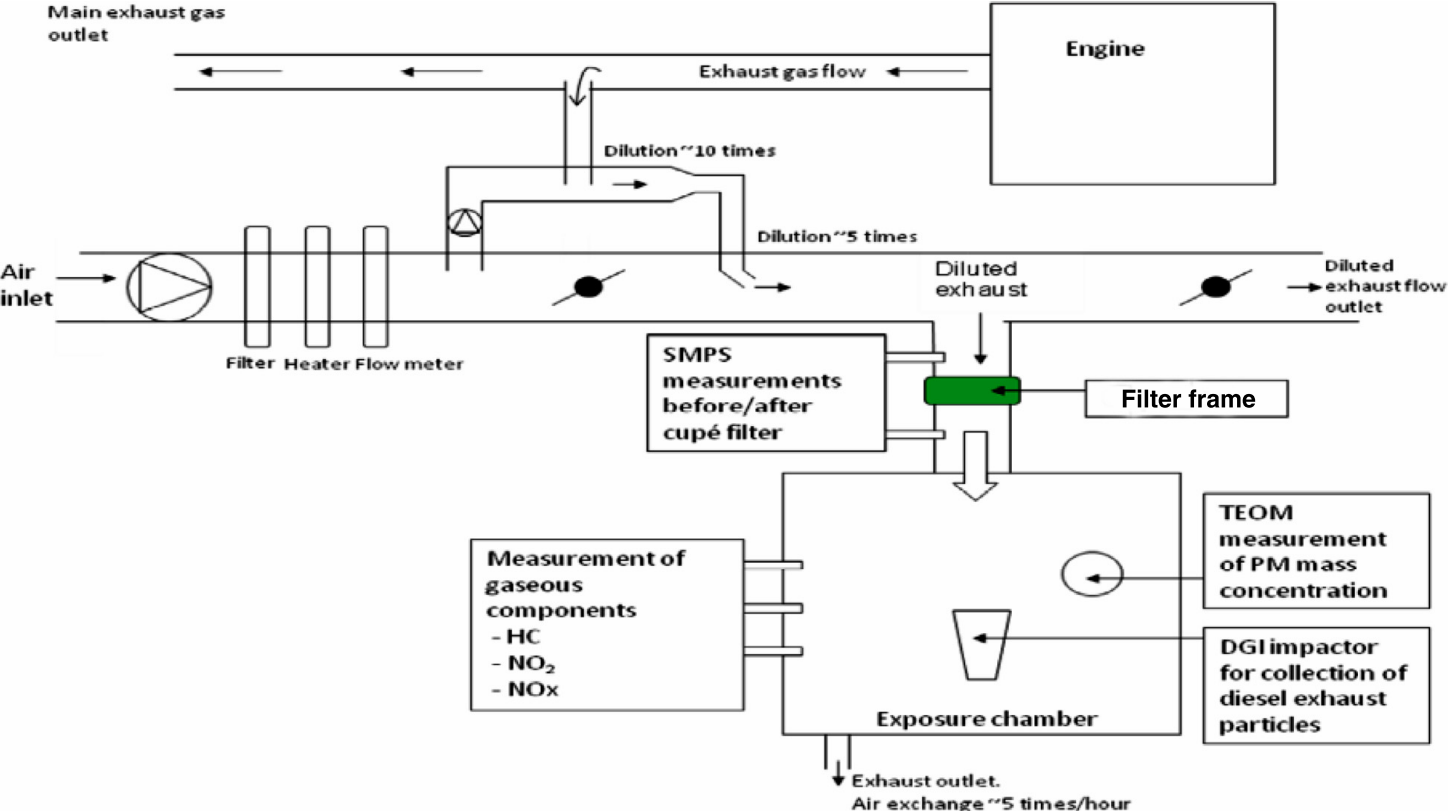
**Schematic of the experimental exposure set-up.** In the tubing to the exposure chamber a frame allowed for filters to be placed for filtering of the diluted diesel exhaust flow that was directed to the exposure chamber.

Diesel exhaust was generated by an idling Volvo diesel engine (Volvo TD40 GJE, 4.0 L, four cylinders, 1996) running on a well-characterized diesel fuel (Preem, UN 1202, VSD 10) as previously described [33] and outlined in detail within the Additional file 2. As shown in Figure 1, the tested filters were located prior to the airflow entering the chamber, in a similar way as in our earlier work [31]. During the occasions with filtered air and unfiltered diesel exhaust exposures the test filters were removed from the cabinets, thus enabling the same air/exhaust flow conditions with and without the studied filters in operation. The tubing immediately before and after the filter, which was located immediately before the air entered the chamber, allowed for continuous control of pressure changes across the filters as well as SMPS measurements. For the two filters employed for the human exposure studies, the particle number size distributions in the chamber determined by a scanning mobility particle sizer (SMPS) ranged from 0.014 to 0.660 μm (mobility diameter) with a bimodal distribution with peaks at around 20-40 nm (nucleation mode) and at 100-130 nm (accumulation mode) similar to our previous studies and as shown in Additional file 3: Figure S2 [34]. The SMPS system consisted of a differential mobility analyzer (DMA, TSI model 3071, TSI Inc., United States) and a condensation particle counter (CPC, TSI model 3010, TSI Inc., United States).

### Outcome measures

The primary aim of the current study was to evaluate symptoms following exposure to filtered and un-filtered diesel exhaust in human subjects. The impact of passing the diesel exhaust aerosol through the filters on measures of systemic inflammation and lung function responses in humans *in vivo represented* secondary analysis. General subjective symptoms of well-being (headache, eye irritation, nasal irritation, unpleasant smell, throat irritation, bad taste, nausea, cough and difficulty in breathing) were assessed by questionnaire before and every 15 minute throughout the duration of the exposure, with scores based on a modified Borg scale, ranging from no symptoms (ranked 0) to maximal symptoms (ranked 11) [35]. Spirometry with determination of FEV_1_ and FVC was performed before and one hour after each exposure using a Jaeger MSC Spirometer (Germany). Blood samples were obtained at baseline and 5 hours after exposure. The samples were centrifuged at 3,000 *g* for 30 min at 4°C before plasma was removed and frozen at-80°C for further analysis. Plasma samples were analysed for markers of acute inflammation: Interleukin-6 (IL6), tumor necrosis factor-alpha (TNFα), P-selectin, soluble Intercellular Adhesion Molecule-1 (sICAM-1), and cluster of differentiation 40 ligand (CD40L) using DuoSet ELISA kits (R&D Systems, Abingdon, UK), according to the manufacturer’s instructions. IL-8 was not measured in peripheral blood as previous investigations have shown its usefulness for evaluating lung responses reflected in bronchial biopsies and bronchoalveolar lavage, but not in blood.

### In vitro-testing of the PM penetrating into the chamber

Four filters, including the two selected for the human exposure campaign, were analysed regarding influence on oxidative and inflammatory potential. Details of their filtering capacity, PM sampling, extraction and analysis of oxidative and inflammatory potentials are summarized in the Additional file 2 and Additional file 4. Briefly, PM oxidative potential was assessed in a synthetic respiratory tract lining fluid (RTLF) model at a particle concentration of 50 _m_g/ml. PM oxidative potential was expressed as the percentage loss of ascorbate and glutathione form this model over a 4 hour incubation (pH 7.4, 37°C) as previously described [36,37].

The human type II alveolar epithelial cell line A549 (ATCC CCL-185) was cultured in RPMI 1640 medium (Gibco BRL, Paisley, UK) supplemented with 10% fetal calf serum (FCS, HyClone, Perbio Science, Aalst, Belgium) and 50 μg/mL gentamicin. All cells were maintained at 37°C in a humidified atmosphere with 5% CO_2_. For experiments, cells were seeded in 24-well culture plates at 5 × 104 cells per well and allowed to attach overnight before particle stimulation. Stock solutions of particles were generated at a concentration of 5 mg/mL in a solution of 0.0004% dipalmitoyl lecithin (DPL; Sigma) in distilled water and sonicated for 4 × 1 min, with vortexing in between. Particles were diluted in cell culture medium without FCS or supplements and used at 10, 30, 50 or 100 μg/cm^2^. After a 24-hour incubation the cell free supernatants were harvested and the concentrations of IL-8 were measured using DuoSet ELISA kits (R&D Systems, Abingdon, UK). After removal of supernatants, the cells were washed with PBS and visually given a viability score, assessing cells as either normal, slightly inhibited, growth inhibited or dead.

The decision of which filters that should be included in the human exposure study was based on their filtering efficacy, in-vitro study data and our experiences from the preceding cabin air filter study [32]. While filter D had the highest particle filtration capacity, it lacked an active charcoal component, which previously had been shown to be of importance to significantly reduce symptoms in human subjects exposed to diesel exhaust [31]. Therefore filter B, which was a particle filter with active charcoal and high filtrating capacity, was selected as most promising. Filter A, which was the same filter but without charcoal, was included as reference.

### Statistics

Normality was tested using the Shapiro-Wilk test. Symptom score and inflammatory markers were expressed as medians with inter-quartile range, with lung function and air pollutant data presented as means ± standard deviation. The Wilcoxon Signed-Rank test was used for calculations of differences between the delta changes (maximal minus pre-exposure symptom score) across the exposures. McNemar´s Chi-square test was performed to analyze the difference between the number of subjects reporting symptoms after exposure to unfiltered and filtered DE. The Wilcoxon Signed-Rank test was used to compare the delta change in inflammatory markers in the peripheral blood (change in concentration at 5 hours after exposure minus before exposure) across the exposures. The delta changes in lung function across exposures (one hour after exposure minus before exposure) were compared using paired-sample Student’s *t* Test. The primary comparisons were done between filtered and unfiltered DE. A secondary comparison was done between the two filters examined.

Oxidative capacity of unfiltered and filtered diesel exhaust particles was analysed using the Kruskal-Wallis One-way-analysis of variance (ANOVA) with post hoc analysis with the Games-Howell test for groups of unequal size and variances. Pro-inflammatory effects of unfiltered and filtered diesel exhaust particles were analysed using One-way analysis of variance (ANOVA) with Dunnett’s Multiple Comparison Test. To compare the efficacy of filter A-D with unfiltered DEP, a two-way ANOVA with Tukey’s multiple comparison test was performed. P values of < 0.05 were considered significant.

Data were analysed using SPSS (SPSS Inc. Chicago, IL, USA, version 17) and GraphPad Prism (GraphPad Software, version 5 for PC).

## Results

### Exposure details

During the unfiltered DE exposure the mean PM concentrations within the chamber was 350 μg/m^3^, with NO and NO_2_ concentrations of 2.49 and 0.68 ppm, respectively (Table 1). Filter A was found to reduce the average PM_1_ mass concentration by 47% (P < 0.001) and the particle number concentration by 36% (P = 0.01). Filter B, which contained an active charcoal component, was even more effective, reducing PM_1_ mass by 74% (P < 0.001), with an associated reduction in particle number by 75% (P = 0.001) (Table 1). The combination filter also reduced the concentrations of NO_2_ by 85% (P < 0.001), whilst Filter A showed no significant effect on NO_2_. Hydrocarbon concentrations were not significantly reduced with filter A, but filter B reduced concentrations by an average of 58% (P < 0.001).

**Table 1:** Pollutant concentrations in the exposure chamber during exposure to filtered air, as well as filtered and unfiltered diesel exhaust challenges

**Exhaust component**	**Filtered air (n = 30)**	**Unfiltered DE (28)**	**Diesel exhaust filter A (n = 29)**	**Diesel exhaust filter B (n = 28)**
PM_1_ (μg/m^3^)	4.6 ± 1.7	350 ± 72	183 ± 18***	93 ± 16***
Particle number x 10^5^/cm^3^	n/a	54 ± 10	30 ± 6*	15 ± 3**
NO (ppm)	0.00 ± 0.00	2.49 ± 1.01	2.21 ± 0.66	1.77 ± 0.55**
NO_2_ (ppm)	0.00 ± 0.00	0.68 ± 0.29	0.58 ± 0.20	0.10 ± 0.04***
Hydrocarbons (ppm)	0.00 ± 0.00	2.41 ± 0.81	1.85 ± 0.60*	1.02 ± 0.11***

Data are given as mean ± SD. T-tests were performed to analyze differences in pollutant concentrations between unfiltered DE and DE exhaust after the use of the cabin filters. P values of < 0.05 were considered significant: *P < 0.05, **P < 0.01, ***P < 0.001.

### Symptomatic responses

All of the subjective symptoms examined, eye and nasal irritation, bad smell and taste, plus headache, were experienced in an increased proportion of the subjects following the unfiltered DE challenge compared with filtered air.

**Figure 2:**
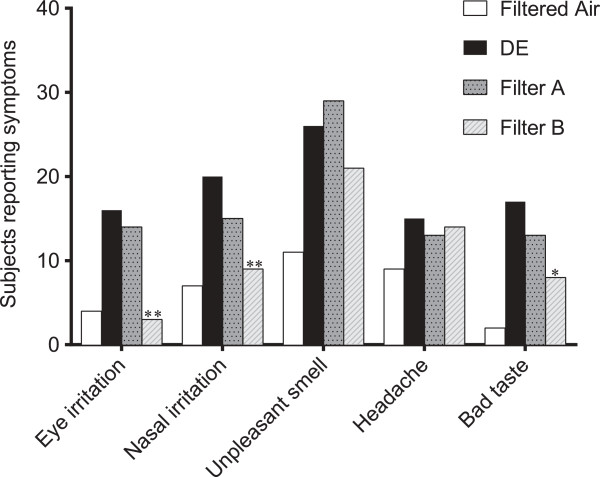
**Number of subjects reporting symptoms after exposure to filtered air, unfiltered diesel exhaust (DE), diesel exhaust with filter A (ultrafine particle filter) and with filter B (ultrafine particle filter with active charcoal).** McNemar´s Chi-square test was performed to analyze the difference between the number of subjects reporting symptoms after exposure to unfiltered DE and diesel exposure filtered with filters A and B. Data were considered significant at ^*^P < 0.05, ^**^P < 0.01

Figure 2 displays the number of subjects reporting symptoms after each exposure. Filter B significantly reduced the number of subjects reporting eye irritation, nasal irritation and bad taste compared with unfiltered DE (p < 0.05-0.01).

**Figure 3:**
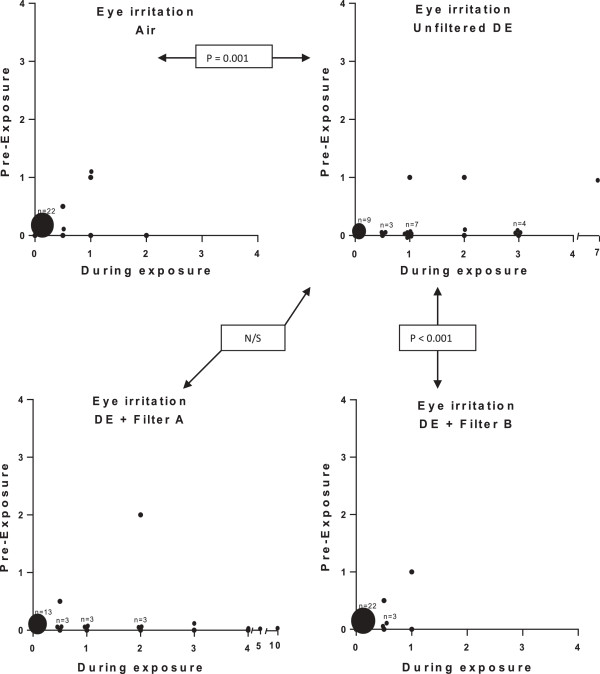
**Graphical illustration of the perception of eye irritation during exposure to filtered air, unfiltered diesel exhaust, and diesel exhaust filtered with Filter A and B, respectively.** The symptom score is presented both pre- and post-exposure according to a modified Borg scale. A shift toward the right indicates increased symptoms during exposure. Each individual is represented by one data point. Where data from several subjects are clustered, the number (n) of subjects is indicated. Wilcoxon’s Signed-Rank test was used for comparison of absolute delta values (Max symptom score minus before exposure) across exposure to unfiltered DE and filter air, unfiltered DE and DE filtered with filter A and unfiltered DE with DE filtered with filter B (active charcoal containing filter). Data were considered significant at p < 0.05. Significant differences between air and unfiltered diesel exhaust, and diesel exhaust filtered with filter A (particle filter) and B (particle and active charcoal filter) are given in the figure. DE with filter B gave significantly less symptom than filter A.

**Figure 4:**
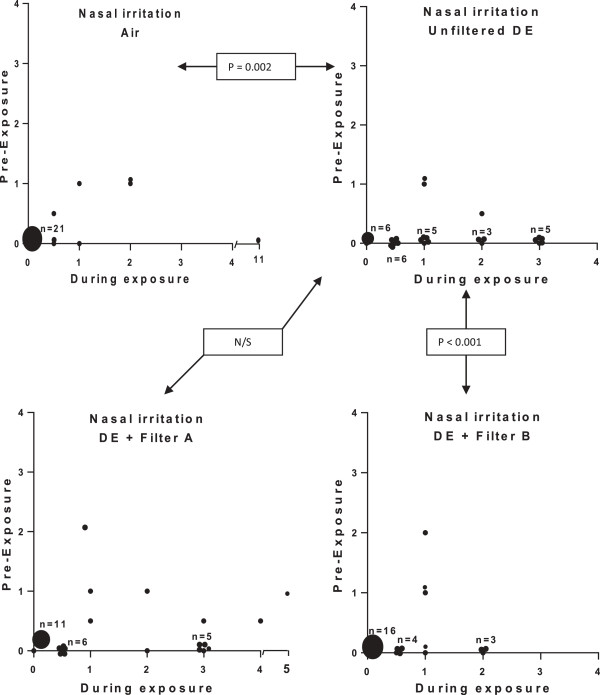
**Graphical illustration of nasal irritation during exposure to filtered air, unfiltered diesel exhaust, and diesel exhaust filtered with Filter A and B, respectively.** Details of the figure and statistical analysis are as outlined in the legend to Figure 3.

**Figure 5:**
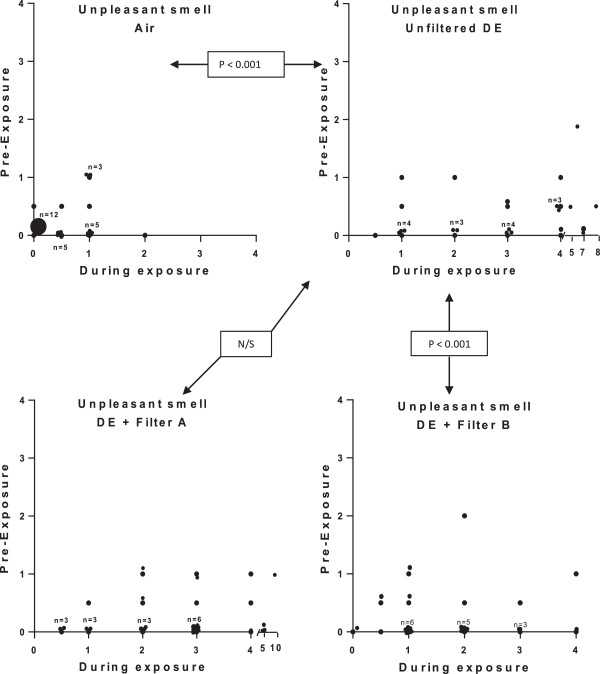
**Graphical illustration of unpleasant smell during exposure to filtered air, unfiltered diesel exhaust, and diesel exhaust filtered with Filter A and B, respectively.** Details of the figure and statistical analysis are as outlined in the legend to Figure 3.

A more detailed description of the influence of symptoms by the different exposures, on an individual subject level, is given in Figures 3, 4 and 5. Eye irritation (Figure 3), nasal irritation (Figure 4) and unpleasant smell (Figure 5) were confirmed to be significantly reduced after exposure with diesel exhaust filtered with filter B (combined particle and active charcoal filter) compared with unfiltered exhaust (p = 0.002-<0.001, Wilcoxon). Only a few subjects experienced eye irritation before and after the filtered air exposure, the proportion increasing to 18 out of 28 following the unfiltered diesel exhaust challenge. Filter B was associated with a significant (P < 0.001) 80% reduction in eye irritation. A similar pattern was seen with nasal irritation, with unfiltered diesel exhaust eliciting symptoms in 20 out of 28 subjects reducing significantly (P < 0.001) to only 6/28 after inclusion of filter B in the exhaust outlet. Filter A did not appear to significantly affect the individual symptom perception. A secondary analysis was performed examining the efficacy to reduce symptoms between Filters A and B. Filter B was significantly more effective in reducing eye irritation, nasal irritation and unpleasant spell and bad taste than filter A (p < 0.01, p < 0.01, p < 0.01 and p < 0.02, respectively.

### Respiratory function test

Exposure to unfiltered diesel exhaust was not associated with a significant reduction in any of the Spirometric variables examined (Table 2). When the delta responses in FEV_1_ (post minus pre-values), of each of the unfiltered and filtered diesel challenges were examined, there was evidence of a statistical reduction (P = 0.035) in the FEV_1_ response with DE, plus filter B, versus unfiltered DE, but the difference was small, of no clinical significance and difficult to interpret in the absence of a DE-induced decrement in the forced expiratory variables.

**Table 2:** Spirometry data before and 1 hour after exposure to filtered air, unfiltered diesel exhaust (DE) and diesel exhaust filtered by filter A and B

	**Filtered air**			**Unfiltered DE**			**DE + filter A**			**DE + filter B**		
	**(n = 30)**			**(n = 28)**			**(n = 29)**			**(n = 28)**		
	**Before**	**1 h after**	**Delta**	**Before**	**1 h after**	**Delta**	**Before**	**1 h after**	**Delta**	**Before**	**1 h after**	**Delta**
FEV_1_	4.27 ± 0.66	4.23 ± 0.63	-0.43 ± 0.19	4.30 ± 0.60	4.23 ± 0.58	-0.04 ± 0.1	4.30 ± 0.64	4.27 ± 0.62	-0.02 ± 0.09	4.24 ± 0.64	4.25 ± 0.64	0.01 ± 0.95*
FVC	5.56 ± 1.01	5.48 ± 0.99	-0.07 ± 0.19	5.60 ± 0.91	5.49 ± 0.94	-0.6 ± 0.16	5.62 ± 0.90	5.60 ± 0.93	-0.2 ± 0.26	5.59 ± 0.97	5.52 ± 0.97	-0.05 ± 0.17
FEV_1_/FVC	77.45 ± 6.29	77.0 ± 6.80	0.24 ± 2.9	77.29 ± 6.33	78.23 ± 7.39	0.72 ± 2.91	76.84 ± 6.70	76.84 ± 6.71	-0.00 ± 3.54	75.74 ± 6.08	77.57 ± 6.10	1.8 ± 3.82

Data are given as mean ± SD. Paired sample T-test was used to compare means of delta changes (1 hour post minus pre exposure) between exposure to unfiltered DE and filter air, unfiltered DE and DE filtered with filter A, and unfiltered DE with DE filtered with filter B (active charcoal containing filter). A p-value of < 0.05 was considered significant. *P < 0.05.

### Systemic markers of inflammation

No changes in the concentration of any of the markers of systemic inflammation were observed when responses across the filtered air and unfiltered DE exposure were compared (Table 3). Again when the responses across each of the DE exposures were compared there was evidence of a significant reduction in the sICAM-1 responses with both DE passed through filter A (P = 0.040) and B (P = 0.026) compared with the unfiltered DE exposure, but again the response was minimal and difficult to explain in the absence of a clear systemic response with the unfiltered DE.

**Table 3:** Inflammatory markers before and 5 hours after exposure to filtered air, unfiltered diesel exhaust (DE) and diesel exhaust filtered by filter A and B

	**Filtered air**			**Unfiltered DE**			**Diesel exhaust + filter A**			**Diesel exhaust + filter B**		
	**(n = 28)**			**(n = 28)**			**(n = 28)**			**(n = 28)**		
	**Before**	**5 hours after**	**Delta**	**Before**	**5 hours after**	**Delta**	**Before**	**5 hours after**	**Delta**	**Before**	**5 hours after**	**Delta**
**IL-6**	0.60	0.68	-0.06	0.64	0.77	0.1	0.69	0.73	-0.03	0.65	0.69	0.04
pg/ml	0.38-0.96	0.43-1.01	-0.16-0.15	0.34-1.01	0.49-1.02	-0.09-0.3	0.64-0-97	0.54-1.04	-0.15-0.22	0.39-0.86	0.4-1.22	-0.19-0.26
**TNF-α**	0.69	0.55	-0.08	0.72	0.61	-0.02	0.88	0.91	-0.05	0.72	0.65	-0.11
pg/ml	0.48-0.93	0.25-0.86	-0.25-0.02	0.42-1.05	0.24-1.00	-0.25-0.05	0.55-1.24	0.39-1.09	-0.25-0.07	0.45-0.97	0.28-1.16	-0.19-0.08
**p-selectin**	69.8	64.7	-0.11	60.0	62.6	1.23	64.4	69.0	-1.98	66.6	66.6	2.52
ng/ml	54.4-80.1	56.7-74.2	-12.7-10.15	45.9-76.1	49.9-77.2	-3.88-10.0	55.9-86.7	54.8-74.1	-17.1-10.8	53.1-83.5	54.9-87.45	-17-4-8.6
**s-ICAM-1**	251	260	3.72	245	244	9.29	265	257	-0.93	298	297	-0.83
ng/ml	208-299	223-312	-16.7-58.5	193-278	214-297	-19.1-42.76	220-329	211-327	-42.4-18.9*	244-318	227-318	-30.1-22.8*
**CD40L**	246	268	29.86	270	244	-13.23	332	249	-11.36	316	251	-78.98
pg/ml	181-331	191-367	-70.8-96.1	173-379	182-320	-99.4-42.3	215-389	159-346	-122.7-48.1	179-434	135-359	-167.3-20.7

Data were expressed as medians with inter-quartile range. Wilcoxon’s Signed-Rank test was used for evaluation of differences between delta changes (5 hours post minus pre exposure) across exposure to unfiltered DE and filter air, unfiltered DE and DE filtered with filter A and unfiltered DE with DE filtered with filter B (active charcoal containing filter). P values of < 0.05 were considered significant. *P < 0.05.

### In vitro measurements

We also examined the oxidative and inflammatory potential of both the unfiltered DE PM and the PM penetrating the filters to reach the subjects in the chamber. These data are provided in the online supplement (Additional file 5: Figure S4 and Additional file 6: Figure S5). The oxidative potential, assessed by the capacity of the sampled PM to drive the oxidation of ascorbate and glutathione from a simple synthetic respiratory tract lining fluid, was low for all PM samples and was not affected by passing the PM through either of the filters tested when examined on a per unit mass (per μg) basis (Additional file 5: Figure S4). The DE particles collected after passing through Filter A, C and D significantly increased IL-8 production from A549 cells. In contrast DE particles filtered by Filter B, which included activated charcoal, did not cause any significant increase in IL-8 release vs. diluent control and the IL-8 levels at the 30, 50 and 100 μg/cm^2^ concentrations were significantly lower than for Unfiltered DE (P < 0.05-0.0001). Cell viability was scored visually and up the concentration 30 ug/cm^2^ the cells were unaffected by all particle samples. At higher particle concentrations unfiltered DE and particles from filter A had the highest effects on cell viability. Filter B with active charcoal showed less cytotoxic effects (Additional file 6: Figure S5).

## Discussion

In this study we demonstrated that the use of an air cabin inlet filter, especially when combined with an active charcoal component, effectively reduces exposure to PM, NO_x_ and hydrocarbons from freshly-generated diesel engine exhaust, improves symptoms and reduces the adverse effect of DE in healthy volunteers, along with decrease in the oxidative and pro-inflammatory effects of the particles which do penetrate the filter. The widespread adoption of such effective air cabin filters has the potential to significantly improve cardiovascular and respiratory health.

### Evaluation of cabin air filters by diesel exhaust exposures in human subjects

Filters A and B were selected for the human exposure study. These filters share the same effective filtering medium, but filter B additionally contains an active charcoal component. The addition of active charcoal to a filter has previously been shown to be beneficial to reduce symptoms following exposure to diesel exhaust [31].

Exposure to traffic-derived air pollution in general and diesel exhaust in particular, has been widely associated with adverse respiratory as well as cardiovascular health effects [6,8]. As a consequence, there is a need to explore different strategies to protect exposed individuals, as expressed by Brook et al in an American Heart Association statement [20]. In this study we have evaluated one possible strategy to reduce PM exposure for commuters and car occupants, given the evidence that this PM exposure in day-to-day life is significantly related to adverse health effects [38].

We demonstrated that the use of an air cabin inlet filter, especially when combined with an active charcoal component, was highly effective to reduce exposure to PM, NO_x_ and hydrocarbons from engine exhaust; with evidence that the combination filter also modified the toxicity of the filtered particles making them less pro-inflammatory in-vitro after filtration. When considering the ability of filters for vehicle cabins to reduce health effects, there are two main properties that are of consideration; 1) the ability to filter particles which is dependent on the mesh and structure of the filter and 2) the ability to filter gaseous components, which depends on adsorption. A partial reduction of particles only may not necessarily be sufficient on its own. We previously addressed this in a bronchoscopy study in human subjects, in which an exhaust pipe filter reduced the particle mass in the exposure chamber roughly by half, but this was insufficient to diminish the diesel exhaust-induced airway inflammatory responses [39]. Similarly, a partial reduction of diesel exhaust particles by an air inlet filter in a preceding study [31] failed to significantly reduce symptoms, whereas the addition of an active charcoal filter component improved the filtering and significantly reduced symptoms. Active charcoal has in the present as well as previous investigations been confirmed to adsorb gaseous components such as NO_2_ and hydrocarbons [31]. There is an extensive literature, which suggests that the pro-inflammatory properties of particles are dependent on the particles’ physical characteristics in combination with their chemical properties [40,41]. Hydrocarbons in diesel exhaust, such as aliphates and PAHs, induce substantial pro-inflammatory effects and mitochondrial dysfunction and we have previously documented these chemical entities to be adsorbed by active charcoal filters [31]. In the present study, the combination filter B including active charcoal, reduced hydrocarbons by half compared with no effect by a particle filter only, and was also superior in reducing both particle number and mass by as much as 75%. The improved particle filtering capacity is expected to be due to the higher adsorption capacity by a filter that includes extensive carbon surfaces, which interacts with particles with hydrocarbons on their surface.

We also found support for the interaction between diesel exhaust particles and the active charcoal to be of importance. In the in-vitro experiments, we studied the release of IL-8 from lung epithelial cells exposed to diesel particles. In human experimental studies, employing bronchoscopy with bronchoalveolar lavage and bronchial mucosal biopsies, we have demonstrated a key pro-inflammatory role for this chemokine in eliciting a neutrophilic airway inflammation through the activation of EGFR, MAPkinase and NFkB pathways [12-14,42-44]. In order to study the toxicological characteristics of the diesel exhaust particles filtered with the various filter media, we chose to expose the cell cultures on an equal particle mass basis. This means that the epithelial cells were cultured with similar particle concentrations, irrespective of the filters actual particle-filtering efficacy. Notably, the combination filter with active charcoal was the only filter to clearly reduce the pro-inflammatory IL-8 release from the cells into the culture medium. It should be noted however that within the exposure context, whilst the inflammatory potential was reduced per unit mass compared with the unfiltered condition, the total inflammatory potential reflects the multiple of the activity, plus the actual exposure concentration and therefore this effect was unlikely to influence the in vivo response.

Pro-inflammatory effects were investigated in-vivo in venous blood samples, as a reasonably accessible means to study systemic inflammation in a large scale multiple exposure study, even if it is acknowledged that bronchoscopy sampling, or invasive measures of cardiovascular function, would have given a substantially more detailed view of the effects of DE and the possibility to intervene with filters. The filters in the human in-vivo study were found to cause a modest though statistically significant reduction in S-ICAM1. This could potentially indicate a protective effect on the vasculature and cell migration from the blood stream by the filtering, however, the absence of any significant effect by unfiltered DE vs. filtered air suggests some caution over the interpretation of the finding.

Oxidative stress has been indicated to be an important mechanism contributing to adverse health effects by air pollutants, such as ozone and particles from different sources. In earlier research we have shown diesel exhaust exposure in human subjects to cause oxidative stress, as reflected in bronchoaolveolar lavage fluid [14,32,45]. We screened the unfiltered and filtered diesel exhaust particles for oxidative potential, which mimics the interaction of particles with respiratory tract lining fluid (RTLF) but were unable to demonstrate any enhanced or reduced oxidation by either the unfiltered or filtered particles, suggesting that the fresh diesel exhaust has low intrinsic oxidative potential. This contrasts with the high oxidative potential measured at roadside sites with high diesel traffic [46] suggesting that other traffic related sources or processes, such abrasion or aerosol ageing may be important.

In accordance with our previous studies [31], exposure to unfiltered DE increased symptoms such as headache, eye irritation, nasal irritation, unpleasant smell, throat irritation and bad taste. Most of these symptoms were significantly reduced by applying filter B, which included active charcoal. This filter was furthermore significantly better than filter A, which contained the same filter medium but was lacking the efficient charcoal component. The mild headache observed in some subjects was less influenced by this filter compared to most other symptoms, suggesting that there could be room for even further improvements of filter technology. The positive effects of using filter technology for reducing DE-related symptoms and the considerable additional effect of adding an active charcoal component is generally in line with our preceding vehicle air cabin study [31]. It should be noted that the diesel exhaust exposure levels in these studies where symptoms were significantly reduced by filtering, have been confirmed to cause respiratory inflammatory effects as well as cardiovascular adverse effects, reflected as vasomotor dysfunction, impaired endogenous fibrinolysis and ST-T segment changes in healthy and subjects with stable coronary heart disease [12,15,47,48]. Reducing symptoms may therefore be accompanied by maintained cardiorespiratory health, which may be of value while driving or travelling in a vehicle, especially in dense and stagnant traffic in metropolitan areas.

### Limitations in the study

In a short-term experimental study, it is only possible to evaluate acute effects, while in real life situations, long-term exposure to traffic-derived air pollution results in increases in cardiovascular and respiratory morbidity and mortality. We have previously demonstrated invasive cardiovascular measurements as well as bronchoscopy evaluation of oxidative and pro-inflammatory effects in the lungs to be powerful tools to study outcomes related to acute adverse effects of traffic exposure [17,47,48]. Studies with such techniques are extremely resource demanding and are very difficult to perform with multiple exposures and interventions, but are powerful as has been shown in some studies [18,31]. The decisions were therefore taken to study the interventive effects of the filters in-vitro with a subsequent human experimental study with non-invasive measurements. The sample size was calculated based on a preceding study [31] but the study population could in an ideal world have been vastly larger, which could have compensated for some unexpected variability in the degree of the responses. Extending the observation and sampling time to 24-72 hours could potentially have given additional information, especially as regards systemic inflammation. However, the complexity of the study would have been vastly increased. The same applies for lung function measurements, where standard spirometry was mainly included as a safety measure, whereas repeated measurements of airway resistance in a body box during and after exposures have previously been shown to be more sensitive to detect airway constriction.

## Conclusions

The present study has confirmed that cabin air inlet filters have the capacity to substantially reduce exhaust-derived particles penetrating into the vehicle cabin. When an efficient filter is combined with an active charcoal component the filtering capacity is enhanced and gaseous components like nitric oxides and hydrocarbons, which have been linked with health effects, are substantially reduced. The combination filter substantially reduced symptoms in human subjects exposed to diesel exhaust and may therefore reduce the adverse health effects for both drivers and passengers. Whilst this solution might be optimal for vehicle users, it is essential that improvements in cabin filters, go hand-in-hand with reductions in tailpipe emissions and other regulations to limit the exposure of the population to traffic emissions.

## Competing interests

This study was commissioned by Renault and funded by means of an unrestricted research grant. Renault had no involvement in the analysis of data or interpretation of the results presented in this paper. S. Couderc and A. Bion are employed by Renault Technocentre, Guyancourt, France.

## Authors’ contributions

AM took part in study design, was responsible for coordinating the study, performed the data and statistical analysis and drafted manuscript. SC and ABI participated in study design and were responsible for filter characterization. ABU, MS and CÖ carried out in vitro inflammatory cell studies. ISM was responsible for the in vitro oxidative characterization of the particles. CB was involved in particle characterization. JP was responsible for determination of inflammatory markers in in vivo studies. JAB, AFB, JPL, ABL, ISM and TS participated in study design, interpretation of data and drafting of manuscript. All authors read and approved the final manuscript.

## Supplementary Material

Additional file 1: Figure S1 Flowchart of the human exposure study. Click here for file

Additional file 2 Online supplement. Click here for file

Additional file 3: Figure S2 Particle number concentrations measured by the SMPS in unfiltered diesel exhaust (DE) immediately prior to the exposure chamber, and after filter A and B, respectively. The distributions are given as average distributions with standard deviations. Click here for file

Additional file 4: Figure S3 Prestudy test data on filtering efficacy in relationship to PM size. Courtesy of Renault. Iso A2 (coarse & fine particles) & NaCl (ultra fine particles) based tests. Click here for file

Additional file 5: Figure S4 Ascorbate concentrations remaining in a synthetic RTLF following a 4 h incubation with 50 g/ml diesel exhaust particles generated from a diesel engine operating under idling conditions, with and without post exhaust filtering with a variety of cabin filters (A-D). Data are illustrated as means (SD) of between 1-7 separate filters for each condition, with each filter analyzed in triplicate. C0 = the time zero ascorbate concentration; C4 = the concentration of ascorbate after the 4 h incubation in the particle free control; CB = the negative control carbon black particle. Click here for file

Additional file 6: Figure S5 Release of IL-8 from alveolar A549 cells following in-vitro instillation with diesel exhaust particles (DEP) generated from a diesel engine without filtering or with filters A-D. Filter B was a combination filter, which included active charcoal. Cells were incubated with medium alone or with 10, 30, 50 or 100 μg/cm2 of particles (n = 4). The level of IL-8 in 24 h supernatants was assessed by ELISA, and expressed as pg/104 cells ± SD vs. untreated cells. One-way ANOVA with Dunnett’s post hoc test was performed to compare with untreated cells. Data were considered significant at *P < 0.05, **P < 0.01, ***P < 0.001. Two-way ANOVA with Tukey´s post hoc test was used to compare IL8 release data between unfiltered DEP and Filters A-D. Data were considered significant at †P < 0.05, ††P < 0.01, †††P < 0.001, ††††P < 0001. Cell viability is given in the upper right panel as unaffected (OK), growth inhibited or dead. Click here for file
