# Supplementary figures and images for: Assessment of the capacity of vehicle cabin air inlet filters to reduce diesel exhaust-induced symptoms in human volunteers
Source: Environ Health. 2014 Mar 13;13:16. doi: 10.1186/1476-069X-13-16 (PMC4007775; doi:10.1186/1476-069X-13-16)

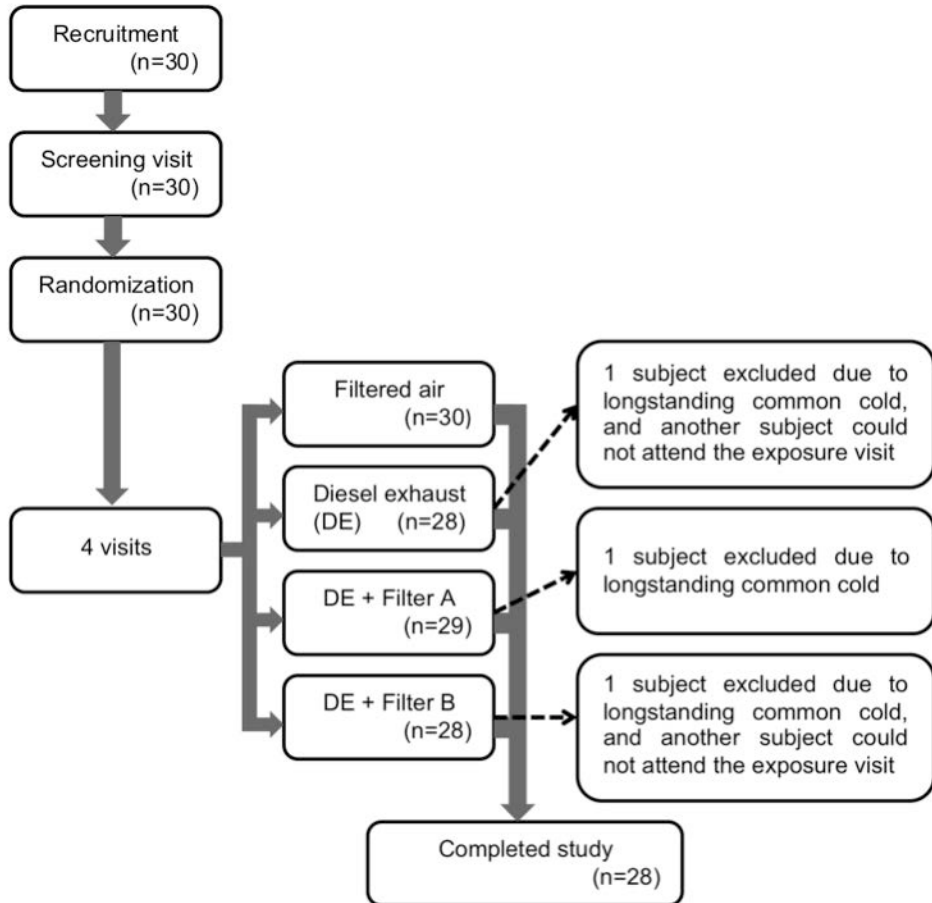

Supplement: Additional file 1: Figure S1 — Flowchart of the human exposure study. [file 1476-069X-13-16-S1.pdf]

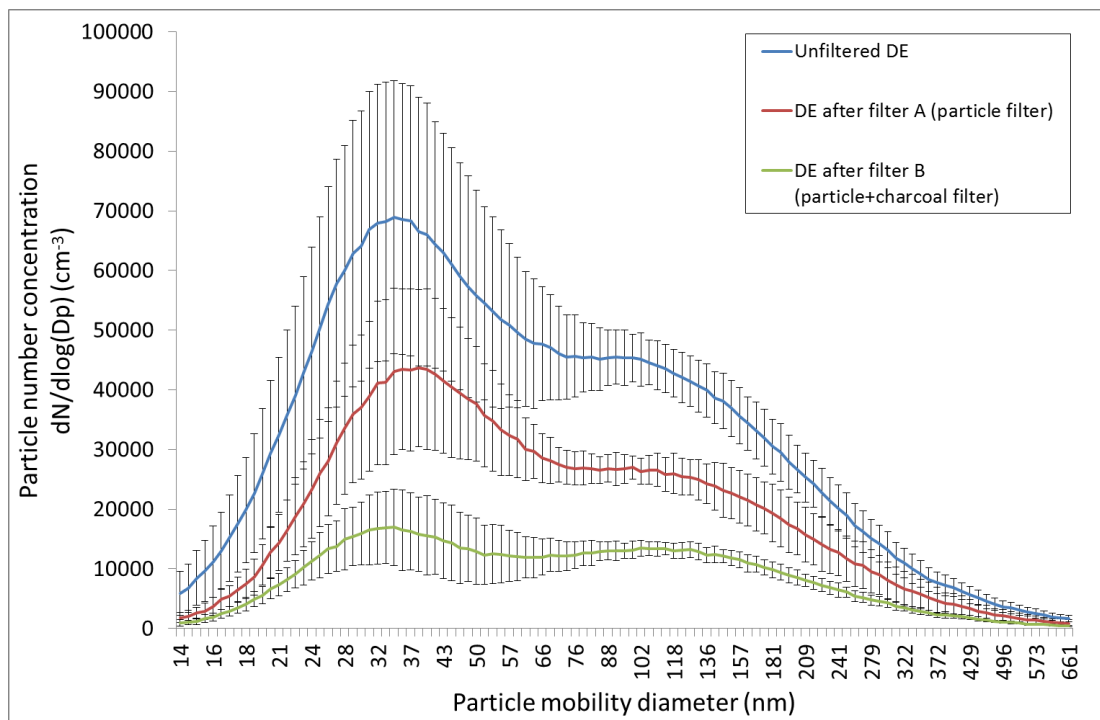

Supplement: Additional file 3: Figure S2 — Particle number concentrations measured by the SMPS in unfiltered diesel exhaust (DE) immediately prior to the exposure chamber, and after filter A and B, respectively. The distributions are given as average distributions with standard deviations. [file 1476-069X-13-16-S3.pdf]

# Particle Collection Efficiency

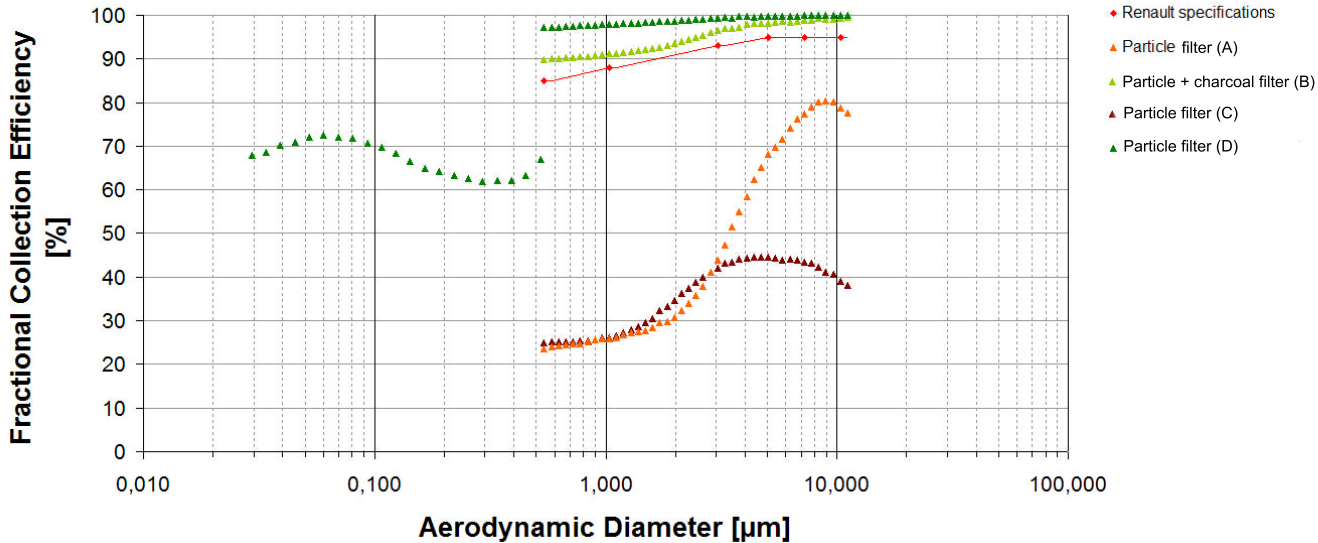

Supplement: Additional file 4: Figure S3 — Prestudy test data on filtering efficacy in relationship to PM size. Courtesy of Renault. Iso A2 (coarse & fine particles) & NaCl (ultra fine particles) based tests. [file 1476-069X-13-16-S4.pdf]

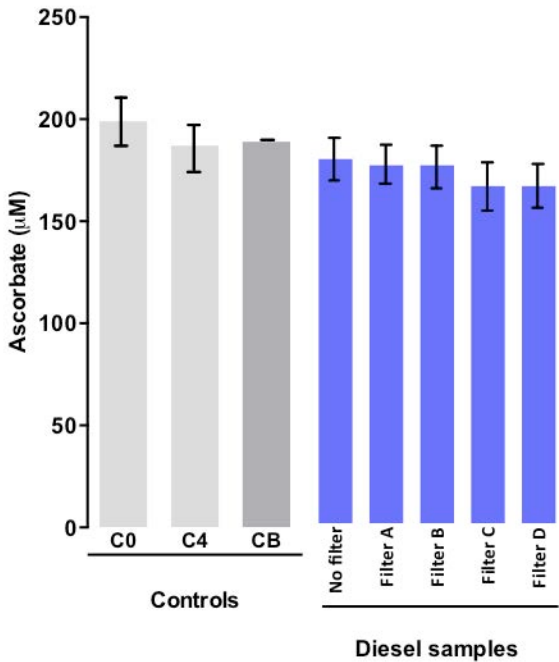

Supplement: Additional file 5: Figure S4 — Ascorbate concentrations remaining in a synthetic RTLF following a 4 h incubation with 50 g/ml diesel exhaust particles generated from a diesel engine operating under idling conditions, with and without post exhaust filtering with a variety of cabin filters (A-D). Data are illustrated as means (SD) of between 1-7 separate filters for each condition, with each filter analyzed in triplicate. C0 = the time zero ascorbate concentration; C4 = the concentration of ascorbate after the 4 h incubation in the particle free control; CB = the negative control carbon black particle. [file 1476-069X-13-16-S5.pdf]

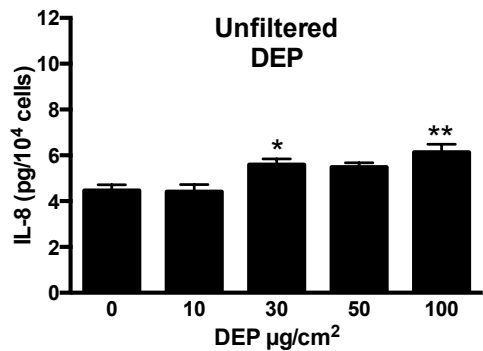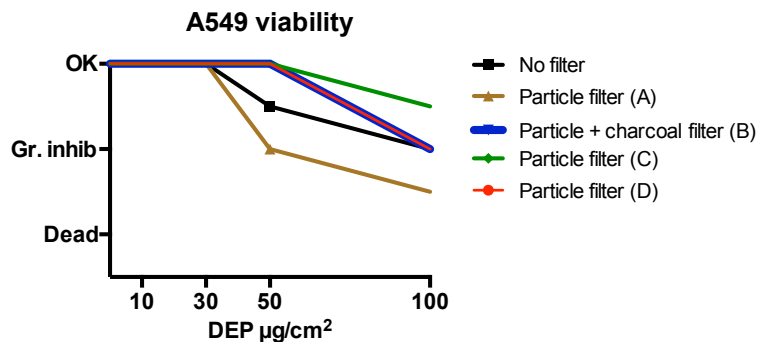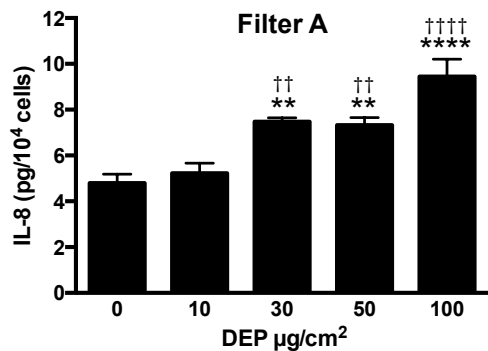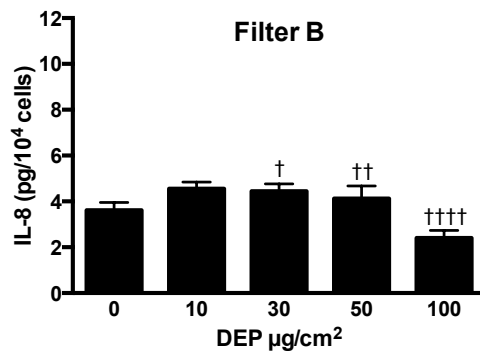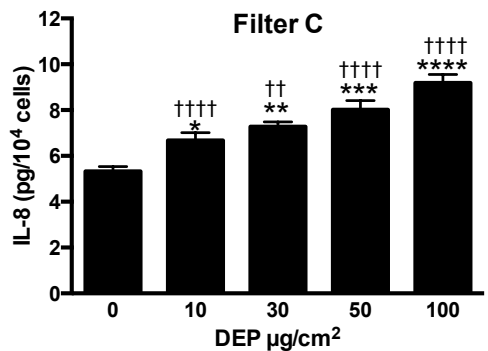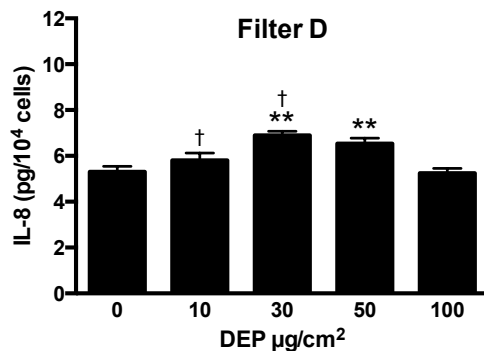

Supplement: Additional file 6: Figure S5 — Release of IL-8 from alveolar A549 cells following in-vitro instillation with diesel exhaust particles (DEP) generated from a diesel engine without filtering or with filters A-D. Filter B was a combination filter, which included active charcoal. Cells were incubated with medium alone or with 10, 30, 50 or 100 μg/cm2 of particles (n = 4). The level of IL-8 in 24 h supernatants was assessed by ELISA, and expressed as pg/104 cells ± SD vs. untreated cells. One-way ANOVA with Dunnett’s post hoc test was performed to compare with untreated cells. Data were considered significant at *P < 0.05, **P < 0.01, ***P < 0.001. Two-way ANOVA with Tukey´s post hoc test was used to compare IL8 release data between unfiltered DEP and Filters A-D. Data were considered significant at †P < 0.05, ††P < 0.01, †††P < 0.001, ††††P < 0001. Cell viability is given in the upper right panel as unaffected (OK), growth inhibited or dead. [file 1476-069X-13-16-S6.pdf]
