# Supplementary material for: Assessment of the capacity of vehicle cabin air inlet filters to reduce diesel exhaust-induced symptoms in human volunteers
Source: Environ Health. 2014 Mar 13;13:16. doi: 10.1186/1476-069X-13-16 (PMC4007775; doi:10.1186/1476-069X-13-16)
Supplement: Additional file 2 — Online supplement. [file 1476-069X-13-16-S2.docx]

**ONLINE SUPPLEMENT**

*The following material is suggested to be presented as an online supplement, for the interested reader who would like more details, rather than being placed in the main manuscript.*

**EXPANDED EXPOSURE METHODOLOGY**

**Diesel generation and aerosol characterization:** Diesel exhaust was generated by an idling Volvo diesel engine (Volvo TD40 GJE, 4.0 L, four cylinders, 1996) running on a well-characterized diesel fuel (Preem, UN 1202, VSD 10). A partial flow of the diesel exhaust was diluted with HEPA-filtered air and after conditioning fed into an exposure chamber at a steady state concentration, as described previously [1, 2]). The main gaseous components of the diesel exhaust were continuously monitored within the chamber using standard real-time instruments for NO/NO_2_ (by chemiluminescence)_,_ CO (by infrared spectroscopy) and total hydrocarbons (by flame ionization). A tapered element oscillating microbalance (TEOM 1400, Rupprecht & Patashnick Co., Inc., United States) equipped with a PM_10_ inlet was used for on-line monitoring of particle mass concentration. In addition, collections of PM for mass determinations and subsequent toxicological assessment were performed using a five-stage gravimetric impactor (DGI 1570, Dekati Ltd, Tampere, Finland). The DGI was adjusted prior to the campaign by removing stages 3-5, thus only including the two top stages as well as the backup filter. This configuration enabled separation of the complete fine mode (<1 µm) PM on the backup filter, while coarse PM (>1 µm) was collected on the upper two stages. The backup filter with fine exhaust-related PM was used for subsequent toxicological analysis as described below. In the upper two stages, PTFE filters (Ø=47 mm) were used (Millipore, Fluoropore membrane filters, 0.2 µm FG, Cat No. FGLP04700), while PTFE filters (Ø=70 mm) were used as backup filters (Millipore, Fluoropore membrane filter, 1.0 µm FA, Cat No. FALP09050). The DGI flow rate used was 90 L/min. After sampling, backup filters were frozen at -20°C prior to extraction. The particulate matter was extracted from the filters with methanol using vortexing only. This was sufficient to recover in excess of 90% of the PM mass, as described previously (ref, Mudway 2004) . The extracted PM was utilized to evaluate the oxidative capacity and pro-inflammatory activity of the unfiltered and filtered DE.

**In-vitro assessment of PM properties:** Unfiltered DE PM, plus PM_10_ passed through four different cabin filters, including the two used in this study for the human challenges were collected for toxicological assessment. The filters, A-D (supplied by Renault and sized 250x200x30mm) were located before the exposure chamber to mimic the filter position in the outside-air intake system of a vehicle´s passenger compartment. PM oxidative activity was assessed in a well validated synthetic respiratory tract lining fluid (RTLF) model on an equal mass basis (50 μg/mL), as previously described in detail [3, 4]. This evaluated the capacity of the PM suspensions to deplete ascorbate and glutathione from the RTLF (each at a starting concentration of 200 μM) over a 4 h incubation period (37^o^C).

After the 4h incubation period, samples were centrifuged for 60 minutes at 13,000 rpm (4oV) prior to either traetment with metaphosphoric acid to a final concentration of 5% (w/v) for UA and AA measurement or diluted in 100 mM phosphate buffer for the determination of GSH. The PM oxidative potential of unfiltered and filtered diesel exhaust particles was analysed using the Kruskal-Wallis One-way-analysis of variance (ANOVA) with post hoc analysis with the Games-Howell test for groups of unequal size and variances.

**REFERENCES**

1. Barath S, Mills NL, Lundback M, Tornqvist H, Lucking AJ, Langrish JP, Soderberg S, Boman C, Westerholm R, Londahl J *et al*: **Impaired vascular function after exposure to diesel exhaust generated at urban transient running conditions**. *Part Fibre Toxicol* 2010, **7**:19.

2. Lucking AJ, Lundback M, Barath SL, Mills NL, Sidhu MK, Langrish JP, Boon NA, Pourazar J, Badimon JJ, Gerlofs-Nijland ME *et al*: **Particle traps prevent adverse vascular and prothrombotic effects of diesel engine exhaust inhalation in men**. *Circulation* 2011, **123**(16):1721-1728.

3. Steenhof M, Gosens I, Strak M, Godri KJ, Hoek G, Cassee FR, Mudway IS, Kelly FJ, Harrison RM, Lebret E *et al*: **In vitro toxicity of particulate matter (PM) collected at different sites in the Netherlands is associated with PM composition, size fraction and oxidative potential--the RAPTES project**. *Part Fibre Toxicol* 2011, **8**:26.

4. Godri KJ, Harrison RM, Evans T, Baker T, Dunster C, Mudway IS, Kelly FJ: **Increased oxidative burden associated with traffic component of ambient particulate matter at roadside and urban background schools sites in London**. *PLoS One* 2011, **6**(7):e21961.

**Figure Legends for online supplement figures**

**Figure s1:** Prestudy test data on filtering efficacy in relationship to PM size. Courtesy of Renault. Iso A2 (coarse & fine particles) & NaCl (ultra fine particles) based tests.

Figure s2: Flowchart of the human exposure study.

Figure s3. Particle number concentrations measured by the SMPS in unfiltered diesel exhaust (DE) immediately prior to the exposure chamber, and after filter A and B, respectively. The distributions are given as average distributions with standard deviations.

Figure s4. Ascorbate concentrations remaining in a synthetic RTLF following a 4h incubation with 50 μg/ml diesel exhaust particles generated from a diesel engine operating under idling conditions, with and without post exhaust filtering with a variety of cabin filters (A-D). Data are illustrated as means (SD) of between 1-7 separate filters for each condition, with each filter analyzed in triplicate. C0 = the time zero ascorbate concentration; C4 = the concentration of ascorbate after the 4h incubation in the particle free control; CB = the negative control carbon black particle.

Figure s5. Release of IL-8 from alveolar A549 cells following in-vitro instillation with diesel exhaust particles (DEP) generated from a diesel engine without filtering or with filters A-D. Filter B was a combination filter, which included active charcoal. Cells were incubated with medium alone or with 10, 30, 50 or 100 µg/cm2 of particles (n = 4). The level of IL-8 in 24 h supernatants was assessed by ELISA, and expressed as pg/104 cells ± SD vs. untreated cells. One-way ANOVA with Dunnett’s post hoc test was performed to compare with untreated cells. Data were considered significant at *P<0.05, **P<0.01, ***P<0.001. Two-way ANOVA with Tukey´s post hoc test was used to compare IL8 release data between unfiltered DEP and Filters A-D. Data were considered significant at †P<0.05, ††P<0.01, †††P<0.001, ††††P<0001. Cell viability is given in the upper right panel as unaffected (OK), growth inhibited or dead.
